# mHealth Intervention for Vietnamese Living With Diabetes: Protocol for a Stepped Wedge Pilot Study

**DOI:** 10.2196/48585

**Published:** 2023-09-28

**Authors:** Anna Nguyen, Zsolt Nagykaldi, Thanh Bui, Sixia Chen, Michael Businelle, Valerie Eschiti, Kathleen Dwyer

**Affiliations:** 1 Fran and Earl Ziegler College of Nursing University of Oklahoma Health Sciences Center Oklahoma City, OK United States; 2 Department of Family and Preventive Medicine University of Oklahoma Health Sciences Center Oklahoma City, OK United States; 3 Hudson College of Public Health University of Oklahoma Health Sciences Center Oklahoma City, OK United States

**Keywords:** Vietnamese, type 2 diabetes, diabetes self-management, mobile health technology, intervention study, stepped wedge design, mobile health, intervention, mobile app, digital health, diabetes, self-management, awareness, mhealth, implementation

## Abstract

**Background:**

Evidence indicates participation in a diabetes self-management education and support program improves self-care behaviors and hemoglobin A_1c_. Language and cultural differences may be barriers to program participation resulting in ineffective self-management, but these factors can be addressed with appropriate interventions. Given the high health care costs associated with diabetes complications, we developed a multicomponent, culturally tailored Self-Management Mobile Health Intervention for US Vietnamese With Diabetes (SMart-D).

**Objective:**

This study aims to evaluate the SMart-D intervention’s feasibility, acceptability, and effectiveness with intentions to scale up the intervention in the future. This mixed methods study incorporates the Reach, Effectiveness, Adoption, Implementation, Maintenance framework to evaluate the intervention.

**Methods:**

This stepped wedge randomized controlled pilot study will be conducted over 2 years in collaboration with primary care clinics. Eligible participants are patients with type 2 diabetes who are receiving health care from participating clinics. Clinics will be randomly assigned to an implementation date and will begin with patients enrolling in the control period while receiving standard care, then cross over to the intervention period where patients receive standard care plus the SMart-D intervention for over 12 weeks. Focus groups or interviews will be conducted with clinicians and patients after study completion. Qualitative data will be analyzed using NVivo. Outcomes on self-care behavior changes will be measured with the Summary of Diabetes Self-Care Activities scale and clinical changes will be measured using laboratory tests. A generalized linear mixed-effect model will be used to compute time effect, clustering effect, and the interaction of the control and intervention periods using SAS (version 9.4; SAS Institute).

**Results:**

We hypothesize that (1) at least 50% (n=5) of eligible clinics and 50% (n=40) of eligible patients who are invited will participate, and at least 70% (n=56) of patients will complete the program, and (2) patients who receive the intervention will have improved self-care behaviors and clinical test results with at least 75% (n=60) of the patients maintaining improved outcomes at follow-up visits compared with baseline, and participants will verbalize that the intervention is feasible and acceptable. As of August 2023, we enrolled 10 clinics and 60 patients. Baseline data results will be available by the end of 2023 and outcome data will be published in 2025.

**Conclusions:**

This is the first Vietnamese diabetes self-management education and support intervention that leverages mobile health technology to address the barriers of language and culture differences through collaboration with primary care clinics. This study will provide a better understanding of the implementation process, demonstrate the potential effectiveness of the intervention, accelerate the pace of moving evidence-based interventions to practice among the US Vietnamese population, and potentially provide a replicable implementation model that can be culturally adapted to other non-English speaking ethnic minorities.

**International Registered Report Identifier (IRRID):**

DERR1-10.2196/48585

## Introduction

Diabetes is a leading cause of death in the US Vietnamese population [1]. The term US Vietnamese will be used hereafter to indicate Vietnamese individuals living in the United States. Members of racial and ethnic minority groups are at an increased risk for developing type 2 diabetes (T2D) and experiencing complications from this disease [2]. After being diagnosed with T2D, individuals face many physical, emotional, and social challenges including the need to know about the strict diet and medication regimen and necessary lifestyle changes [3].

Nutrition is often the focus of self-management among US Vietnamese with diabetes, and little focus is placed on medication, blood glucose monitoring, risk reduction behaviors, or personal strategies for health promotion [4-6]. Self-management and health behaviors are influenced by cultural beliefs, practices, and availability of resources [7]. Ineffective self-management can lead to complications and increased health care costs. Glycemic control can reduce the risks of diabetes complications, but few resources are available to educate and support self-management among US Vietnamese. Despite the capability of improving health behaviors with evidence-based interventions, efforts to adapt and disseminate linguistic and culturally appropriate diabetes self-management education and support (DSMES) are lacking among this group.

Nguyen and Edwards [6] reported that linguistic and cultural differences as well as transportation issues are the main barriers to participating in existing DSMES programs among study participants. Many US Vietnamese living with T2D receive information from family, friends, and others diagnosed with diabetes for knowledge and decision-making around self-care [6]. Without formal DSMES, knowledge gained from informal education may negatively influence self-care behaviors. Low diabetes literacy hindered effective disease self-management, which is a striking finding among the participants that was revealed in every interview [6]. A needs assessment survey identified the community’s desire and need for formal diabetes education and support to be offered in the Vietnamese language in Oklahoma [8]. Studies showed that having limited English proficiency reduced the ability of study participants to obtain appropriate information to manage chronic conditions, such as heart disease and diabetes [9,10].

The 2022 National Standards for Diabetes Self-Management Education and Support [11] emphasized the need to provide services that embrace cultural differences, address social determinants of health, and use technological engagement platforms and systems. These National Standards cited strong evidence supporting DSMES delivery through digital, telehealth, telephone, SMS text messaging, and web-based or mobile phone app [11].

Nguyen et al [12] conducted a systematic review of intervention studies focused on improving health behaviors and health care services using mobile technology among the Vietnamese population and found the main device used was mobile phones. These devices were used for patient remote monitoring, assessment, or counseling for health behavior change through SMS text messaging, internet access, email, and videos. SMS text messaging was most frequently used as the primary intervention, which is consistent with many studies that have evaluated the usage of mobile health (mHealth) to support lifestyle and health behavior changes in other populations [13-16]. Using SMS text messaging to provide health education and monitor chronic diseases was feasible and acceptable mainly due to the low cost and easy operation of SMS text messaging [12]. However, the feasibility and acceptability of SMS text messaging to provide education and support among US Vietnamese patients is unknown and thus the aim of this study’s protocol.

In response to the burden of diabetes and the need to afford an opportunity for US Vietnamese with T2D an intervention that meets the National Standards [11], this Self-Management Mobile Health Intervention for US Vietnamese With Diabetes (SMart-D) study protocol will provide linguistically and culturally relevant diabetes education, health behavior change support, and emotional support through secure mHealth technology to help patients understand the disease process and gain skills to manage their condition. This protocol aims to describe a pilot study to test the acceptability and feasibility of the SMart-D intervention, determine the effectiveness, and assess its sustainability using the Reach, Effectiveness, Adoption, Implementation, Maintenance (RE-AIM) framework to measure outcomes. Focus groups or individual interviews will be conducted at program completion to assess intervention acceptability and participant experiences as well as perceived barriers to sustaining the behavior change.

## Methods

### Study Design

This study is a stepped wedge randomized controlled pilot study to implement a multicomponent intervention among US Vietnamese with T2D. The stepped wedge design allows the practical staggering of intervention delivery while enabling all participants to receive it. The intervention, incorporating both synchronous and asynchronous components over 12 weeks, is based on the patient empowerment and Health Belief Model and intends to target the unique needs of Vietnamese-speaking patients. Participants will receive a print booklet covering self-management strategies, daily mHealth SMS text messaging to promote self-care and enhance self-efficacy, and weekly nurse calls to coach and provide support for self-management goals. Offering these ongoing contacts will help to motivate participants to follow the care plan and engage in better self-management.

After the 12-week intervention, participants will opt to accept or decline a low dose of 1 monthly SMS text message and 1 monthly nurse call alternating biweekly for the next 9 months. This study will evaluate pre- and postintervention measures at 3, 6, 9, and 12 months from baseline. This framework will allow a comparison of outcomes between the standard care and intervention period as well as between baseline and follow-up data. To ensure the high quality of this pilot, this study will follow the SPIRIT (Standard Protocol Items: Recommendations for Interventional Trials) 2013 checklist [17,18].

### Intervention Components

The intervention has 3 components: print materials, mHealth SMS text messages, and nurse coaching telephone calls. Print education materials and follow-up telephone calls have been shown to be incrementally effective on their own [19-23]. Combining these components along with mHealth SMS text messaging may achieve a more significant effect. All 3 intervention components were assessed for usage and usability by piloting the components with 2 nurses and 4 Vietnamese individuals with T2D. Modifications to the intervention components were made based on the participants’ feedback.

All participants will receive a copy of printed materials entitled Small Change, Big Impact for People with Diabetes, which covers 8 chapters focusing on patient-centered strategies and incorporating cultural foods and practices. The content focuses on the day-to-day activities of living with diabetes with respect for the US Vietnamese individual’s life experiences helping participants (1) understand diabetes, (2) monitor blood glucose, (3) stay physically active, (4) cope with the diagnosis, (5) make healthy food choices, (6) understand Eastern and Western medications, (7) manage hypo- or hyperglycemia, and (8) prevent diabetes complications. The education content incorporates the Association of Diabetes Care and Education Specialists framework [24] and materials developed by the Centers for Disease Control and Prevention [25] diabetes resources and were adapted for cultural relevancy. All printed materials are available in Vietnamese and English language, side-by-side, to help participants learn the terminologies to communicate with their providers.

An mHealth SMS text message will be used to deliver educational content in the Vietnamese language via video, audio, and SMS text messages based on a schedule when specific criteria are met. The prerecorded videos are embedded in the SMS text messages and feature a registered dietician consulting with participants on developing a healthy eating plan that incorporates Vietnamese food and a pharmacist demonstrating insulin injection and self-monitoring of glucose. The secure mHealth platform will enhance both accurate, timely data collection across sites and ensure secure data transmission. During the 12-week intervention at least 1 message will be sent daily; up to 6 messages may be sent depending on the participants’ responses. If a participant does not respond to messages for 7 consecutive days, the coach nurse will assess any technical issues during the routine weekly calls. After the 12-week intervention, messages will be sent at a lower dose once monthly for the remaining follow-up period to allow continuing engagement with participants as they desire.

Participants will have a telephone coaching call with a Vietnamese-speaking nurse once per week during the 12-week intervention period. The day and time of these phone calls will be arranged between the participant and the nurse. Every week, participants will be encouraged to set one or more goals related to the scheduled lesson. During these coaching calls, the nurse will follow a script and use a motivational interviewing approach [26,27] to review participants’ goals and identify barriers, review status on self-management strategies, help participants build a capacity to problem solve, improve self-efficacy, and support health behavioral change. The use of a motivational interviewing approach by the coach nurse will encourage each participant’s intrinsic motivation to engage in self-care, change health behaviors, and improve their glycemic control [28]. Using a checklist, the nurse will document the content of each call and emphasize key educational topics including discussion of weekly goal attainment, discussion of barriers if the goal is not met, setting of goals for next week’s lesson, and provision of the opportunity to obtain personalized feedback if participants experience any challenges, and address concerns.

### Patient and Public Involvement in Intervention Development

The SMart-D intervention was developed with substantial engagement with the Vietnamese community of Oklahoma, including leaders of the Asian District Cultural Association, faith-based leaders, health care professionals, and people with T2D. This 23-member group served as an advisory board for over a 1-year period and highlighted the need to develop a feasible and appropriate study design as well as the need to incorporate language and culture-relevant information [29]. By incorporating the Health Belief Model, this SMart-D intervention emphasized the need to reduce diabetes complication risks and motivated participants to take into account the central concept of risk, thus encouraging health behavior change [30]. Accordingly, both Vietnamese language and cultural practices are strong components of this intervention, and using the stepped wedge design allowed all consented participants to receive the intervention. In addition, the intervention was preliminarily tested with a group of targeted individuals prior to implementation to ensure the usage and usability of the education content and method of delivery. After this study is completed, reports will be made available to clinics and patients in the form of a newsletter as well as a scheduled in-person seminar where the research team will describe findings with each individual clinic.

### Study Procedures

#### Inclusion or Exclusion Criteria

There are 2 levels of inclusion and exclusion: clinics and patients. Clinics are eligible if they currently serve more than 5 US Vietnamese adults diagnosed with T2D. T2D patients who identify as Vietnamese adults and receive health care at a participating primary care clinic will be eligible. Patients must be 18 years of age or older, diagnosed with T2D, able to provide verbal or written consent in English or Vietnamese, able to speak or read Vietnamese, and possess or have access to a mobile phone.

The exclusion criteria at the clinic level are those clinics that currently serve fewer than 5 US Vietnamese patients with T2D. At the patient level, individuals younger than 18 years of age, individuals visiting the United States as tourists, those with cognitive impairment, those unable to provide consent in English or Vietnamese, and pregnant women are excluded as pregnancy affects diabetes care differently at each trimester [11]. Patients will be terminated early if they become pregnant or explicitly refuse to have blood drawn as ordered by their physicians.

#### Clinic Recruitment and Consent Process

Primary care clinics will be identified according to the geographical area in which many US Vietnamese patients receive health care services. Prior to recruiting clinics of large health care systems, permission will be sought from the responsible administrators and medical directors. Independent and privately owned clinics will receive an invitation letter describing this study and its aims. Clinics that respond to the invitation will be assessed for eligibility by the principal investigator (PI). Clinics that do not respond to the initial mailed letter within 2 weeks will be contacted by email or phone.

Once the clinics are determined to be eligible, the PI will contact the office manager and the health care provider to ask for approval for the clinics to participate and obtain consent from these individuals. Next, the PI will set up 2 sessions of 1 hour briefing about this study and 1 hour training with clinic staff. Then participating clinics will identify and refer interested patients with a diagnosis of T2D via telephone contact or email.

#### Patient Recruitment and Consent Process

This study will recruit 80 patients from 10 clinics, approximately 8 patients from each clinic. We expect to have approximately 5 to 6 patients complete this study from each clinic giving a 70% (n=56) study completion rate. The power calculation was conducted by using the linear mixed effect modeling of the R (R Foundation for Statistical Computing) package “*swdpwr*,” which gives power of 0.95 for the alternative hypothesis treatment effect change of 0.005 (2-sided type I error 0.05). The recruitment strategy includes a letter and flyer to be sent by the clinics with the research team’s contact information. Study information will be provided to those who reach out to a research team member with their interest and an opt-out contact will be applied after 3 days of receiving the letter. A member of the research team will arrange a time to meet with eligible patients to discuss the option to participate in this study, complete the informed consent procedure, complete this study’s screener, and enroll them in this study if the individual is both eligible and interested. All patients will have the option of completing the consent and confidentiality forms in English or Vietnamese language. Upon full study enrollment, a member of the research team will collect baseline data.

#### Randomization

Because individual randomization is not feasible due to potential contamination of the intervention for control participants, this study will use a programmatic roll-out in a stepwise fashion where each clinic that is not yet receiving the intervention will function as a control. Another reason for the selection of a stepped wedge design is that the measurement of outcomes is at the clinic level and individual patient level. Randomization of the intervention sequence will be conducted at the level of the clinics with computer-generated assignment using SAS (version 9.4; SAS Institute) by a statistician blinded to the identity of the clinics and not involved in the intervention delivery. The clinics will be randomized from 1 through 10 and will be allocated a date of implementation which will be concealed until interventions are assigned. Clinics will be informed that the order of allocation to the intervention will be unknown in advance of randomization.

**Figure 1:**
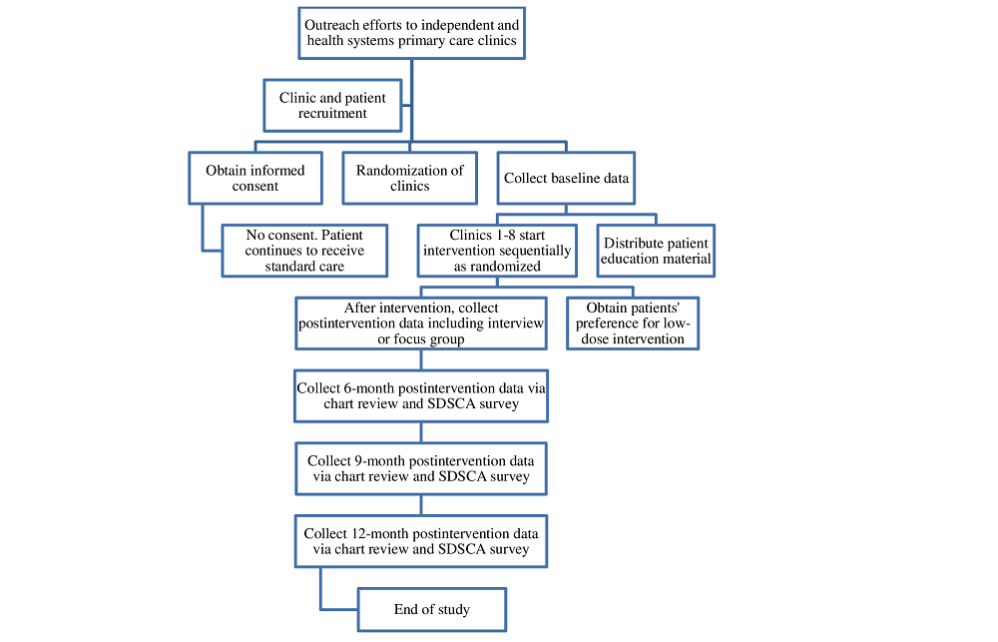
Study procedures for a randomized clinical trial in collaboration with primary care clinics. SDSCA: summary of diabetes self-care activities.

During the initial control period, no clinic will be exposed to the intervention. Subsequently, at each 3-month interval, 1 clinic will transition from the control to the intervention period. This staggered schedule will allow for optimal training, implementation, and supervision of clinic staff. The process will continue until all 10 clinics have crossed over to receive the intervention. Figure 1 illustrates this study’s procedures.

#### Data Collection

At enrollment, participants will complete the self-report demographic information survey, which will include age, gender, years of residence in the United States, and clinical information about their current treatment regimen to characterize the sample. For baseline data, participants will also complete the validated Summary of Diabetes Self-Care Activities (SDSCA) scale to measure self-care behaviors. The SDSCA scale, available in both English and Vietnamese languages, measures the participant’s management of diabetes in the last 7 days regarding diet, physical activity and exercise, blood glucose testing, foot care, and smoking. Participants answer 11 questions by indicating the number of days the behavior was followed in the past 7 days (0-7 days). In addition to self-reported behaviors, lab test results including triglyceride, low-density lipoprotein, high-density lipoprotein, hemoglobin A_1c_, and clinical assessments including BMI, blood pressure, and weight will be collected from their medical chart and entered into Research Electronic Data Capture (Vanderbilt University), a secure database. This information will be collected again at completion of the 12-week program and at 6 months, 9 months, and 12 months from baseline. Table 1 describes the timing of data collection and evaluation plan by the RE-AIM framework.

**Table 1:** Data collection and evaluation plan using RE-AIM^a^ framework: assessment objectives, data, instruments, and timeline.

RE-AIM domain		Method	Baseline	Follow-ups (months after baseline)			
				3	6	9	12
Demographic patient information		Paper questionnaire	✓	N/A^b^	N/A	N/A	N/A
Clinic or facility information		Paper questionnaire	✓	N/A	N/A	N/A	N/A
**Reach: absolute number and characteristics of those participating in the intervention**							
	Percentage of eligible patients who respond to texts and nurse calls at least 1 week of program	mHealth^c^ data and field notes	✓	N/A	N/A	N/A	N/A
	Differential characteristics between participating and nonparticipating patients	Clinic data and field notes	✓	N/A	N/A	N/A	N/A
	Barriers and facilitators to reach	Focus groups or interviews	✓	N/A	N/A	N/A	N/A
**Effectiveness: modification in self-care behavior and improvement in biological markers**							
	Changes in SDSCA^d^ scale	Paper questionnaire	✓	✓	✓	✓	✓
	Changes in triglyceride, LDL^e^, HDL^f^, HbA_1c_^g^, BMI, BP^h^, weight	Clinical assessment and lab results	✓	✓	✓	✓	✓
	Percentage of patients enrolled at end of study period	Enrollment data	✓	✓	✓	✓	✓
**Adoption: intervention adoption at clinic and patient level**							
	Percentage of eligible clinic sites who participated	Enrollment data	✓	N/A	N/A	N/A	N/A
	Barriers and facilitators to adoption	Focus groups or interviews	✓	N/A	N/A	N/A	N/A
**Implementation: key features of program delivery**							
	Fidelity to the protocol	mHealth data and field notes	✓	✓	✓	✓	✓
	Barriers and facilitators to implementation (feasibility and acceptability)	Focus groups or interviews	✓	✓	N/A	N/A	N/A
**Maintenance: extent of clinics sustaining the program and patients maintaining improved outcomes**							
	Sustainability of intervention at each site through end of study period	Focus groups or interviews	✓	✓	✓	✓	✓
	Sustainability of improved outcomes through the end of the study period	Clinical assessments and lab results	✓	✓	✓	✓	✓

^a^RE-AIM: Reach, Effectiveness, Adoption, Implementation, Maintenance.

^b^N/A: not applicable.

^c^mHealth: mobile health.

^d^SDSCA: summary of diabetes self-care activities.

^e^LDL: low-density lipoprotein.

^f^HDL: high-density lipoprotein.

^g^HbA_1c_: glycated hemoglobin.

^h^BP: blood pressure.

#### Outcome Measures

The primary outcomes are the degree of reach, effectiveness, adoption, implementation, and maintenance measured by using the RE-AIM framework [31]. Reach is the ability to recruit patients and will be determined by assessing the number of people who received an invitation over the number of those who may be eligible to participate in the same clinic. Data sources include the number of invitations and the number of eligible patients seeking care at the clinic. Adoption will be measured by the proportion of clinicians who used the program and the number of patients who participate in the intervention by assessing the number of enrolled participants over the number of those invited, 3 months after the initiation of the intervention. Program implementation will be assessed by conducting a focus group or interview with the clinicians and patients separately at each clinic to gather feedback. Differences and similarities will be compared within and across clinics to identify emerging themes. Barriers and facilitators to program adoption by clinics will also be identified. See Table 2 for postintervention focus group or interview questions.

**Table 2:** Postintervention focus group or interview questions for participants.

Measures	Interview questions
Acceptability	How would you describe your experience of taking part in this tailored DSMES^a^ program? What aspect of the program did you like the most? What was your favorite session? Suppose a friend of yours was diagnosed with diabetes and came to you for advice. What would you tell them about this DSMES program?
Process of change	What did you learn from this program? How has your perspective of living with diabetes changed since completing the program? What differences have you noticed in your life as a result of taking part in this DSMES program?
Barriers	What kind of difficulties did you experience taking part in this DSMES program?
Improvement	What did you least like about the program? What do you think could be improved about this DSMES program?
Implementing change	How often do you practice the self-care behaviors such as following a healthy eating plan, testing your blood sugar, caring for your foot, etc. learned from this program?

^a^DSMES: diabetes self-management education and support.

The secondary outcomes are self-reported changes in behaviors and clinical test results, which will be compared to determine intervention maintenance and effectiveness. Repeated outcome measures will be assessed at 5 points in time. The translated and validated SDSCA revised scale [32] will be used to measure behavioral changes and assess 5 topics: diet, physical activity and exercise, blood glucose testing, foot care, and smoking. The average interitem correlations within scales were high (mean *r*=0.47) with the exception of specific diet; test-retest correlations were moderate (mean *r*=0.40). Correlations with other measures of diet and exercise generally supported the validity of the SDSCA subscales (mean *r*=0.23). Triglyceride, low-density lipoprotein, high-density lipoprotein, hemoglobin A_1c_, BMI, weight, and blood pressure will be used to measure clinical changes, which will be obtained from the participants’ medical records. Participants will be monitored for an additional 9 months via chart reviews and self-report using the SDSCA scale to determine whether any improvements observed at completion of the intervention will still be evident.

#### Data Analysis

To evaluate the first hypothesis, descriptive statistics will be used to analyze the acceptability and feasibility of the intervention, which include the proportion of individuals who met the criteria, proportion of eligible participants who agreed to participate, the proportion of mHealth responses completed, and attrition rate at follow-up periods. A linear regression model will be used to examine the association of response rate and attrition rate with other predictors. To evaluate the second hypothesis, continuous variables will be calculated using descriptive statistics including quantiles, mean, and SD. To examine the association of continuous outcome variables and other predictors, a linear mixed effect model will be used to examine time effects, clustering effects, and the interaction of the two. Transformation of outcome variables will be conducted to normalize the nonnormal distributions. To examine the association of categorical outcome variables and other predictors, a generalized linear mixed effect model will be used to examine time effects, clustering effects, and the interaction of the two. In order to handle missing values, sequential multiple imputation will be performed before the analysis. SAS (version 9.4; SAS Institute) will be used for statistical analysis.

To analyze qualitative data from the focus groups and interviews, field notes will be transcribed for coding and analysis. Focus groups and interviews conducted in Vietnamese will be translated into English by the research assistant and verified by the PI before coding and analyzing thematically. Prior to analyzing the transcripts, the researchers will read the transcripts at least twice and compare them with the field notes to ensure accuracy and completeness. Selected transcripts will be reviewed by an experienced qualitative researcher to compare the consistency in developing a coding schema. The NVivo (Lumivero) software will be used to manage and analyze qualitative data.

### Intervention Fidelity

The intervention uses 3 fidelity checks to ensure that participants have received the intended information. First, the mHealth SMS text messages will be distributed automatically. Second, progress will be monitored and followed up with participants who abruptly stop reading or responding to our SMS text messages. Third, the nurse coaching call sessions will be monitored randomly by the PI, who will make note of any deviations and will provide further training for the nurse. These fidelity assessments are structured to ensure the intervention is implemented as intended during the delivery process.

### Ethics Approval

Ethical approval has been granted by the University of Oklahoma Health Sciences Center institutional review board on October 25, 2022 (IRB#14700). Permission from the large health systems and each clinic has also been granted. The SMart-D intervention involves research with an ethnic minority and understudied population. To ensure the needs of this population are met, the research team has led a prior project with members of this community who contributed to the design, development, and implementation of this study. Ethical procedures include steps for continuation of health education for participants after study completion by coordinating with local health departments to refer participants to the existing and structured Total Wellness program of Oklahoma. Baseline data and lessons learned will be presented at conferences, and study outcomes will be published in scientific journals. Study results will also be communicated to relevant health care professionals and to the US Vietnamese community through lectures, posters, and pamphlets.

## Results

Planning for the SMart-D study began in January 2022, clinic recruitment began October 28, 2022, and patient recruitment began November 4, 2022. Data collection of outcome measures will be completed in 2024. This study was funded during July 2022 to June 2024. As of August 24, 2023, 10 clinics and 60 patients were enrolled in this study. The results are expected to be available by 2025 and will demonstrate intervention acceptability, feasibility, effectiveness, and sustainability.

## Discussion

This study is driven by two hypotheses: (1) at least 50% (n=5) of eligible clinics and 50% (n=40) of eligible patients who are invited will participate, and at least 70% (n=56) of patients will complete the program, and (2) patients who receive the intervention will have improved self-care behaviors and improved clinical test results with at least 75% (n=60) of the patients maintaining improved outcomes at follow-up visits compared with baseline, and participants will verbalize that the intervention is feasible and acceptable. This protocol was designed with an interprofessional research group that has many strengths. An overall strength is the stepped wedge design, which allows participating clinics to be randomized with an opportunity for all consented patients to participate in the intervention. The staggering of clinic implementation allows the research team to train clinic staff and monitor potential implementation issues. The mixed method and longitudinal design provide insights into long-term outcomes over time.

Another strength of this protocol is this is the first Vietnamese DSMES intervention to be implemented in collaboration with primary care clinics that leverages mHealth technology to address the barriers of language and cultural differences. Vietnamese-speaking individuals have few opportunities to participate in health-related randomized clinical trials. This protocol allows that opportunity because it is offered in the Vietnamese language to promote self-determination and independence, and improve self-management strategies.

Further, 1 limitation of the stepped wedge design is that patients of clinics that were randomized for implementation later will have to wait months before receiving the intervention. This waiting period may reduce engagement with the researchers and may increase attrition rates. Another limitation is the repeated measurements of questionnaires and clinical tests may lead to a higher respondent burden on patients and clinic staff.

In conclusion, this study will improve understanding of the implementation process, demonstrate the potential effectiveness of the intervention, accelerate the pace of moving evidence-based interventions to practice among the Vietnamese population, and potentially provide a replicable implementation model that can be culturally tailored to other non-English speaking ethnic minorities. Furthermore, successful study results may be implemented across other localities and states and across both primary care and specialty clinics.

## Abbreviations

DSMES: diabetes self-management education and support
mHealth: mobile health
PI: Principle investigator
RE-AIM: Reach, Effectiveness, Adoption, Implementation, Maintenance
SDSCA: Summary of Diabetes Self-Care Activities
SMart-D: Self-Management mobile health Intervention for US Vietnamese with Diabetes
SPIRIT: Standard Protocol Items: Recommendations for Interventional Trials
T2D: type 2 diabetes

## Data Availability

Data sharing is not applicable to this paper, no data sets were analyzed during the development of this protocol. To support scientific transparency, we will publish deidentified data and additional supporting files in the main paper.
